# Supplementary material for: Chronic Use of Proton-Pump Inhibitors and Iron Status in Renal Transplant Recipients
Source: J Clin Med. 2019 Sep 3;8(9):1382. doi: 10.3390/jcm8091382 (PMC6780301; doi:10.3390/jcm8091382)
Supplement: Supplementary file 1 [file jcm-08-01382-s001.pdf]

Supplemental Results

Description of Excluded RTR Receiving Oral Iron Supplementation

In our study, 41 RTR received oral iron supplementation at the time of the study visit and were excluded from our statistical analyses. Baseline differences between RTR with oral iron supplementation and without oral iron supplementation are demonstrated in Table S5. RTR using oral iron supplementation had significantly lower eGFR ($p < 0.001$), serum creatinine levels ($p < 0.001$) and had more often proteinuria ($p < 0.001$). In addition, diuretics were more often used by iron supplement users. Hemoglobin levels of RTR using iron supplementation were significantly lower ($p < 0.001$). PPI use did not significantly differ between iron supplement users compared to non-users. Based on these analyses we do not think that exclusion of these RTR materially affected the outcome of our study.

Supplemental Tables

Table S1. Logistic regression analyses investigating the association of PPI use with ID in 626 stable RTR (H2RA users excluded).

	**Iron Deficiency**		
**n = 626**	**Odds ratio**	**95% CI**	***p***
**Crude**	1.99	1.39 – 2.86	<0.001
**Model 1**	1.99	1.38 – 2.88	<0.001
**Model 2**	1.66	1.12 – 2.47	0.01
**Model 3**	1.67	1.09 – 2.55	0.02
**Model 4**	1.66	1.11 – 2.47	0.01
**Model 5**	1.67	1.12 – 2.48	0.01
**Model 6**	1.50	0.99 – 2.25	0.05

Model 1: PPI use adjusted for age and sex. Model 2: model 1 + adjustment for eGFR, proteinuria, time since transplantation, history of GI-disease. Model 3: model 2 + adjustment for lifestyle parameters (BMI, smoking behavior, alcohol use, dietary iron intake). Model 4: model 2 + adjustment for inflammation (hs-CRP). Model 5: model 2 + adjustment for MMF use. Model 6: model 5 + adjustment for other medication use (diuretic use, RAAS-inhibition, antiplatelet therapy, CNI use and prednisolone use). Abbreviations: CNI, calcineurin inhibitor; MMF, mycophenolate mofetil; RAAS-inhibitors, renin-angiotensin-aldosterone system inhibitors.

Table S2. Logistic regression analyses investigating the association of PPI use with ID (TSAT < 20% and ferritin < 100 µg/L) in 646 RTR.

	Iron Deficiency		
**n = 646**	**Odds ratio**	**95% CI**	***p***
**Crude**	2.90	1.94 – 4.35	<0.001
**Model 1**	2.89	1.92 – 4.35	<0.001
**Model 2**	2.34	1.51 – 3.62	<0.001
**Model 3**	2.44	1.53 – 3.89	<0.001
**Model 4**	2.34	1.51 – 3.62	<0.001
**Model 5**	2.34	1.51 – 3.62	<0.001
**Model 6**	2.15	1.38 – 3.36	0.001

Model 1: PPI use adjusted for age and sex. Model 2: model 1 + adjustment for eGFR, proteinuria, time since transplantation, history of GI-disease. Model 3: model 2 + adjustment for lifestyle parameters (BMI, smoking behavior, alcohol use, dietary iron intake). Model 4: model 2 + adjustment for inflammation (hs-CRP). Model 5: model 2 + adjustment for MMF use. Model 6: model 5 + adjustment for other medication use (diuretic use, RAAS-inhibition, antiplatelet therapy, CNI use and prednisolone use). Abbreviations: CNI, calcineurin inhibitor; MMF, mycophenolate mofetil; RAAS-inhibitors, renin-angiotensin-aldosterone system inhibitors.

Table S3. Logistic regression analyses investigating the effect of medication use on the association of PPI use with ID in 646 RTR.

	**Iron Deficiency**		
**n = 646**	**Odds ratio**	**95% CI**	***p***
**Model 5a**	1.49	1.01 – 2.20	0.05
**Model 5b**	1.57	1.07 – 2.31	0.02
**Model 5c**	1.53	1.04 – 2.25	0.03
**Model 5d**	1.53	1.04 – 2.26	0.03
**Model 5e**	1.57	1.07 – 2.31	0.02

Model 5: model 2 + adjustment for medication use (5a: diuretic use, 5b: RAAS-inhibition, 5c: antiplatelet therapy, 5d: CNI use and 5e: prednisolone use).

Table S4. Logistic regression analyses investigating the association of PPI use with ID (TSAT < 20% and ferritin < 300 μg/L) in 646 RTR.

	**Model 1**		**Model 2**		**Model 3**		**Model 4**		**Model 5**		**Model 6**	
Nr of events = 193	OR (95% CI)	*p*	OR (95% CI)	*p*	OR (95% CI)	*p*	OR (95% CI)	*p*	OR (95% CI)	*p*	OR (95% CI)	*p*
PPI use	1.94 (1.36–2.78)	<0.001	1.57 (1.07–2.31)	0.02	1.57 (1.04–2.38)	0.03	1.56 (1.06–2.30)	0.03	1.57 (1.07–2.31)	0.02	1.43 (0.96–2.12)	0.08
Age, y	1.00 (0.99–1.02)	0.9	1.01 (0.99–1.02)	0.4	1.00 (0.99–1.02)	0.8	1.01 (0.99–1.02)	0.5	1.01 (0.99–1.02)	0.4	1.00 (0.99–1.02)	0.8
Female Sex	1.79 (1.26–2.52)	0.001	1.96 (1.37–2.80)	<0.001	1.88 (1.27–2.78)	0.002	1.95 (1.36–2.79)	<0.001	1.97 (1.38–2.81)	<0.001	1.96 (1.35–2.85)	<0.001
eGFR, mL $min^{-1} \cdot 1.73\ m^2$			0.99 (0.99–1.01)	0.8	0.99 (0.99–1.01)	0.9	1.00 (0.99–1.01)	0.9	0.99 (0.99–1.01)	0.8	1.00 (0.99–1.01)	0.5
Proteinuria			1.78 (1.15–2.76)	0.009	1.93 (1.21–3.07)	0.005	1.73 (1.11–2.69)	0.02	1.78 (1.15–2.75)	0.01	1.88 (1.20–2.93)	0.006
Time since transplantation, y			0.95 (0.93–0.98)	0.8	0.96 (0.93–0.98)	0.002	0.95 (0.93–0.98)	0.001	0.96 (0.93–0.99)	0.006	0.96 (0.93–0.99)	0.04
History of GI-disease			0.92 (0.45–1.89)	0.001	0.94 (0.45–1.98)	0.9	0.91 (0.44–1.88)	0.8	0.92 (0.45–1.86)	0.8	0.92 (0.44–1.91)	0.8
BMI, kg/m^2					1.04 (0.99–1.08)	0.06						
Current smoker					0.46 (0.24–0.88)	0.02						
Alcohol user					0.74 (0.49–1.11)	0.1						
Dietary iron intake, mg/d					0.99 (0.93–1.06)	0.8						
hs-CRP, mg/L							1.03 (1.01–1.06)	0.006				
RAAS-inhibition											0.74 (0.51–1.06)	0.1
Diuretics											1.50 (1.01–2.22)	0.04
Antiplatelet therapy											1.40 (0.89–2.19)	0.1
CNI use											1.64 (1.09–2.48)	0.02
MMF use									1.08 (0.69–1.70)	0.7	1.21 (0.75–1.94)	0.4
Prednisolone use											0.99 (0.90–1.10)	0.9

Model 1: PPI use adjusted for age and sex. Model 2: model 1 + adjustment for eGFR, proteinuria, time since transplantation, history of GI-disorders. Model 3: model 2 + adjustment for lifestyle parameters (BMI, smoking behavior, alcohol use, dietary iron intake). Model 4: model 2 + adjustment for inflammation (hs-CRP). Model 5: model 2 + adjustment for MMF use. Model 6: model 5 + adjustment for other medication use (diuretic use, RAAS-inhibition, antiplatelet therapy, CNI use and prednisolone use). Abbreviations: CNI, calcineurin inhibitor; MMF, mycophenolate mofetil; RAAS-inhibitors, renin-angiotensin-aldosterone system inhibitors.

Table S5. Baseline characteristics of RTR with and without oral iron supplementation.

Characteristics	RTR with iron supplementation	RTR without iron supplementation	*p*
Number of subjects, n (%)	41	646	n/a
PPI use	22 (53.7)	363 (56.2)	0.8
Demographics			
Age, years	51 ± 15	53 ± 13	0.2
Men, n (%)	14 (34.1)	382 (59.1)	0.002
BMI, kg/m^2	25.4 ± 4.5	26.7 ± 4.8	0.09
Diabetes Mellitus, n (%)	8 (19.5)	157 (24.3)	0.5
History of GI–disease, n (%)	5 (12.2)	42 (6.5)	0.2
Time since transplantation, years	6.1 (2.4–13.1)	5.3 (1.8–12.0)	0.6
Lifestyle parameters			
Current smoker, n (%)	5 (13.2)	79 (13.1)	1.0
Alcohol consumer, n (%)	22 (56.4)	409 (70.6)	0.06
Iron intake, mg/d	11.0 ± 2.9	11.3 ± 2.9	0.5
Renal function parameters			
eGFR, ml · min^{-1} · 1.73 m^{-2}	36.6 ± 16.7	53.5 ± 19.9	<0.001
Serum creatinine, µmol/L	160 (125–240)	122 (99–156)	<0.001
Proteinuria (≥0.5 g/24h), n (%)	19 (46.3)	135 (21.0)	<0.001
Laboratory parameters			
Iron deficiency, n (%)	12 (29.3)	193 (29.9)	0.9
Hb, g/dL	12.2 ± 1.5	13.3 ± 1.7	<0.001
Iron, µmol/L	16.5 ± 6.9	15.2 ± 5.9	0.3
Ferritin, µg/L	142.0 (69.5–324.5)	115.5 (53.0–216.3)	0.07
Transferrin saturation, %	29.0 ± 12.7	25.1 ± 10.5	0.06
Glucose, mmol/L	5.1 (4.6–6.2)	5.3 (4.8–6.0)	0.4
HbA1c, mmol/mol	38.2 (34.4–42.3)	39.9 (36.6–44.3)	0.06
HsCRP, mg/L	1.6 (0.9–6.0)	1.6 (0.8–4.2)	0.4
Medication use			
Calcineurin inhibitors, n (%)	25 (61.0)	369 (57.1)	0.6
Mycophenolate mofetil, n (%)	24 (58.5)	431 (66.7)	0.3
Prednisolone, n (%)	40 (97.6)	641 (99.2)	0.3
Diuretics, n (%)	23 (56.1)	253 (39.2)	0.03
RAAS–inhibitors, n (%)	17 (41.5)	314 (48.6)	0.4
Antiplatelet drugs, n (%)	7 (17.1)	131 (20.3)	0.6
H2–receptor antagonists, n (%)	0 (0)	20 (3.1)	0.6

Data are presented as mean ± SD, median with interquartile ranges (IQR) or number with percentages (%). Abbreviations: BMI, body mass index; eGFR, estimated glomerular filtration rate; Hb, hemoglobin; HbA1c, hemoglobin A1c; HsCRP, high-sensitivity C-reactive protein.
